# Validation of activity trackers to estimate energy expenditure in older adults with cardiovascular risk factors

**DOI:** 10.1371/journal.pone.0309481

**Published:** 2024-08-27

**Authors:** Alina Rieckmann, Bas Jordan, Friederike Burczik, Jacqueline Meixner, Christian Thiel

**Affiliations:** 1 Department of Applied Health Sciences, Division of Physiotherapy, Hochschule für Gesundheit, University of Applied Sciences, Bochum, Germany; 2 Faculty of Sport Science, Ruhr University, Bochum, Germany; Manipal Academy of Higher Education, INDIA

## Abstract

**Objectives:**

To compare different types of activity trackers recording physical activity energy expenditure (PAEE) and examine their criterion validity against indirect calorimetry (IC) as the gold standard in adults over 60 years of age with a special focus on women with cardiovascular risk.

**Design:**

Synchronous registrations of PAEE were performed with up to four different devices to determine criterion validity against IC while participants performed a protocol of simulated activities in a laboratory setting.

**Method:**

Thirty-four participants (25 women, 9 men) with at least a light cardiac risk performed a protocol of simulated activities in a laboratory setting (daily living activities, cycle ergometer test). PAEE was simultaneously assessed by IC, two research-grade activity trackers (ActiGraph-wGT3X-BT and Actiheart-4) and two consumer-level activity trackers (OMRON pedometer and Fitbit Charge-3). Tracker-derived PAEE was compared with PAEE calculated from IC descriptively and by Bland–Altman plots.

**Results:**

The ActiGraph (0.7 ± 0.4 kcal/min), the Actiheart (1.1 ± 0.6 kcal/min) and the OMRON (0.8 ± 0.6 kcal/min) underestimated, while the Fitbit (3.4 ± 1.2 kcal/min) overestimated PAEE compared to IC-PAEE (2.0 ± 0.5 kcal/min). The Bland–Altman limits of agreement (LoA) against IC were +0.5/+2.2 kcal/min for the ActiGraph, -0.3/+2.1 kcal/min for the Actiheart, -3.7/+1.0 kcal/min for the Fitbit, and -0.5/+2.9 kcal/min for the OMRON. The magnitude of the deviation varied considerably depending on the activity (e.g. walking, cleaning, cycle ergometer test).

**Conclusions:**

The research-grade activity trackers estimated PAEE with higher validity than the commercially available activity trackers. The partly very wide LoA have to be critically considered when assessing PAEE in the context of health service research, as individual Physical Activity behaviour may be under- or overestimated.

## Introduction

The amount and the intensity of physical activity (PA), or the physical activity energy expenditure (PAEE) as its product, are closely related to cardio-metabolic health. PA is an important outcome, and the promotion of PA plays a major role in cardiac disease prevention and therapy. Due to the complex nature of PA, its accurate measurement is challenging [1]. Ideally, in cardiovascular disease prevention, therapy and rehabilitation, an objective monitoring of PA would allow for accurately differentiating a wide range of PA intensities, identify behavioural patterns and their determinants for personal advice, individualize activity dose and training load, as well as support and mediate self-efficacy [2]. Depending on the design and characteristics, the accuracy and usability of the various options available to capture PA in controlled or free-living environments may differ [3].

The doubly labelled water (DLW) method and indirect calorimetry (IC) provide the gold standard to estimate energy expenditure (EE). Wearable activity trackers may be more cost-effective, and offer better usability [4], while being able to also capture low intensity activities, which are particularly important for older adults with reduced fitness. The ActiGraph accelerometer (ActiGraph LLC, Fort Walton Beach, FL, USA) and the Actiheart combined accelerometer and heart rate monitor (CamNtech Ltd, Cambridge, UK) are most commonly used in PA research [1, 5–7]. Among the consumer-level devices, hip-worn pedometers and smartwatches have been used by individuals to increase PA in several studies, for example following cardiac rehabilitation [8].

The psychometric properties of activity trackers have been tested in evaluation studies to varying degrees. The ActiGraph accelerometer has been validated for older adults in several studies against DLW or IC [9, 10], unlike the Actiheart branched equation model to measure PAEE. There is generally a lack of validation studies for older adults, and especially older women [11, 12].

At individual level, activity trackers may produce substantial measurement errors in PAEE measurement [1]. PAEE prediction equations derived from younger study populations may not be valid for older adults, persons with reduced physical fitness, or cardiac patients [3, 6, 11]. Their PA behaviour is inherently different concerning overall PA level, type of activities, movement characteristics, movement patterns, maximal exercise performance, and resulting PAEE among other factors [3]. Because of the lack of specific validation studies, activity trackers should be used with great caution in these groups [13, 14].

Consumer-level activity trackers are considered even less accurate than the devices used in research [15]. Their algorithms are proprietary and raw data cannot be accessed [6].

Due to the wide range of activity trackers available, it is difficult to understand and overview their advantages and disadvantages [6] when attempting to answer a specific research question relating to a specific population. Limited research has been done to directly compare the validity of these devices in older adults or in a population with cardiovascular risk factors. Overall, there is an urgent need for better information on the validity of different types of activity trackers in populations of older women with an increased cardiac risk or cardiac disease.

### Objectives

The aim of this study is to directly compare different types of activity trackers recording PAEE or EE and examine their criterion validity against IC as the gold standard in adults over 60 years of age, with a special focus on women with cardiovascular risk. The purpose is to inform scientists and field users about the validity of the devices, to rank research-grade and commercially available activity trackers, and to detect suitable devices for further research as well as for use in health and prevention services.

## Methods

Synchronous registrations of PAEE with up to four different measurement devices were performed to determine criterion validity against IC while participants performed a protocol of simulated activities in a laboratory setting.

Participants were recruited between 12 November 2019 and 04 February 2020 at physiotherapy outpatient practices and venues of different community activities, such as choirs, church communities, and activity groups, in the Ruhr area located in the west of Germany, by direct communication or by means of flyers.

Relevant inclusion and exclusion criteria were screened by telephone interview. We included adults over 60 years of age with cardiovascular risk, such as high blood pressure, high blood cholesterol, or high body mass living independently at home. Exclusion criteria were, among other, no medical clearance to participating in the study in case of unclear health status or existing indications for relative contraindication, serious illness, and severe physical immobility (see S1 Table in S1 File for the full inclusion and exclusion criteria). Coronary risk was assessed using the European cardiovascular disease risk assessment model (Systematic Coronary Risk Evaluation, or SCORE) [16]. Initially, we had planned to recruit n = 45 participants at different cardiac disease stages ranging from light or moderate cardiac risk to manifest cardiac disease. However, due to the COVID-19 pandemic situation, the inclusion of participants with cardiac disease had to be cancelled.

To analyse criterion validity during simulated activities, PAEE as simultaneously measured by activity trackers (ActiGraph wGT3X-BT and Actiheart 4) was compared to the PAEE measured by indirect calorimetry (MetaMax^®^ 3B). In a subsample of only women, PAEE was additionally registered with two commercially available activity trackers (Fitbit Charge 3 and OMRON Walking Style IV Step Counter). Before entering into the protocol, participants were asked to fast for at least 3 h and to refrain from exercise on this particular day.

The activity protocol consisted of simulated activities of daily living (part 1) and a cycle ergometer test (part 2), performed in a laboratory of the University of Applied Sciences Bochum (see S2 Table in S1 File for the full activity protocol). Part 1 consisted of resting (11 minutes), walking (6 minutes), stair climbing (2 minutes), and household activities (7.5 minutes). Part 2 started with 25 W on the cycle ergometer, increasing 25 W every 2 minutes, and was stopped at exhaustion or at Borg 15 (from 20) on the Borg rating of perceived exertion scale for participants with cardiac risk. When at least half of the final stage of the ergometer test was accomplished, data measured during the stage were included in the analysis.

All activity trackers were initialized simultaneously using individually measured characteristics before each trial, including weight, height, age, gender, and stride length if it was required. Following the protocol, EE estimates were obtained and calculated from the devices themselves using the associated software.

Indirect calorimetry (Cortex MetaMax^®^ 3B System and MetaSoft^®^ Studio Software, Cortex Biophysics GmbH, Leipzig, Germany) was used as the gold standard. Before each measurement, the MetaMax 3B was calibrated according to the manufacturer’s guidelines (Calibration Manual, CORTEX Biophysik GmbH, Leipzig, Germany). The VO_2_ and the VCO_2_ data were collected with a soft mask over the nose and mouth (breath by breath), and the heart rate was measured (POLAR belt around the chest) to determine the individual oxygen uptake rate and EE. An evaluation of the validity and reliability is available elsewhere [17].

The mean PAEE in kcal/min was calculated from the calorimetry-measured VO_2_ and VCO_2_ for each individual for the periods of resting (11 minutes), walking and climbing stairs (8 minutes), simulated activities of daily living (7.5 minutes), and the ergometer test. The resting metabolic rate was predicted using the Harrington equation [18, 19]:

Female population:

$$Resting\;Energy\;expenditure\;[REE]\;\left(\frac{kcal}{min}\right)=\frac{(BMI*28.15)-(age*6.44)+905}{1440}$$

Male population:

$$Resting\;Energy\;Expenditure\;[REE]\;\left(\frac{kcal}{min}\right)=\frac{(BMI*28.15)-(age-6.44)+1290}{1400}$$

Weir’s equation [20] was used to calculate total energy expenditure (TEE) as follows:

TEE(kcal/min)=3.941xVO2(l/min)+1.106xVCO2(l/min)+1.106xVCO2(l/min)

The PAEE resulted from the subtraction of the REE from the TEE.

Participants wore the three-plane ActiGraph wGT3X-BT (ActiGraph LLC, FL, USA) on a belt on the right hip. Data were saved in 10-second epochs (ActiLife software V6.13.3, ActiGraph LLC, FL, USA). We deliberately chose the Freedson equation [12] to report PAEE in kcal/hour because it has been widely used in research for estimating energy expenditure or categorise physical activity at different intensity levels [10].

The chest-placed Actiheart 4 (CamNtech, Cambridge, GB) uses vertical plane accelerometry (activity as counts per minute, or cpm) and heart rate data from a one-channel electrocardiogram electrode (heart rate above the resting heart rate, or HRaR). Based on 15-second epochs, PAEE was calculated by branched equations [11, 21] included in the Actiheart software (Version 4.0, CamNtech, Cambridge, GB). The following equation for older adults was entered for the group calibrated model [21]:

$$Accelerometry:\;PAEE\left(\frac{\frac{J}{kg}}{min}\right)=(0.21*cpm)+(77*sex)+21$$

$$Heart\;rate:\;PAEE\;\left(\frac{\frac{J}{kg}}{min}\right)=(5.5*HRaR)+(16*sex)+(1.2*HRaR*sex)-94$$

(sex: female = 0)

Resting heart rate was obtained before starting the protocol in a seated position, as the Actiheart device could only be initialized in this position. Sleeping heart rate was estimated as resting heart rate minus 10 heartbeats. For measurement and data analysis, published standard procedures were used [11].

The Walking Style IV Step Counter (OMRON Healthcare UK LTD, Milton Keynes, UK) is a commercially available lightweight pedometer with a 3D sensor. It measures steps, calories, and the distance walked per day. The pedometer was initially set to weight, height and stride length and then placed in the right front pocket of the trousers, as recommended in the manual. Action mode was started and stopped manually immediately before and after the measurement to report steps and calories (PAEE) for the total activity protocol.

The Fitbit Charge 3 (Fitbit Inc., San Francisco, CA, USA) is a smartphone-based wireless fitness monitor worn on the right wrist. It contains a triaxial accelerometer (micro electro mechanical system), an altimeter measuring altitude differences and an optical heart rate tracker (Fitbit User Manual Version 3.4). Prior to the measurement, a user profile was created for the participants in the app. This profile included age, weight and height. To record activity, an activity log was activated on the device. Due to the indoor measurement, no global positioning system (GPS) data were used. After taking the measurement, it was synchronized to the Fitbit app (Version 3.12, Fitbit Inc., San Francisco, CA, USA). As Fitbit reports TEE, PAEE was calculated by subtracting the REE from the TEE. Data is only available for the total activity protocol, the activities are not analysed individually.

Participants’ health, PA status, and PA-related health competence were assessed with a screening questionnaire (Physical Activity Readiness Questionnaire) [22], the Global Physical Activity Questionnaire (GPAQ) [23] and the Physical Activity-Related Health Competence questionnaire [24].

Informed written consent was obtained from the participants before study initiation. The study complies with the ethical principles for medical research outlined in the World Medical Association’s Declaration of Helsinki and was approved by the ethical committee of the German Association for Physiotherapy (ZVK) e.V., Wremen, Germany (Number 2019–06).

All devices were synchronized to the same date and time before initializing. In order to compare the PAEE of the different devices, time filters were set in the software or later in the calculation in Excel for the whole protocol or individual activities. We did not read Fitbit and Omron results for every activity, only EE for all activities.

Guidelines for missing data (“lost data”) were set in cases where there was missing data for more than one continuous minute. In that case, the time period was excluded for data calculation, and the mean score was calculated from all other periods with valid data. If the data loss was less than one minute, the data were linearly interpolated. A heart rate of <30 beats/min was considered missing data.

Tracker-derived PAEE was compared against PAEE calculated from indirect calorimetry descriptively and by Bland–Altman plots [25]. Comparisons between tracker-derived PAEE and the criterion measure were performed using one-way ANOVA’s. If differences were found, paired samples t-tests were used to identify the location of the differences. The level of significance for these analyses was set at p < 0.05.

## Results

In total, 34 older adults (25 women, 9 men) with at least a light cardiac risk were included in the study. Their risk of cardiovascular death (SCORE^16^) within the next 10 years was 2.6% ± 1.1%, with familial predisposition being the most prevalent risk factor (see Table 1).

**Table 1:** Participant characteristics ^a^^,^^b^.

	Total (n = 34)	Women (n = 25)	Men (n = 9)
Age [y]	71.3 ± 5.6	71.3 ± 5.6	71.2 ± 6.0
Weight [kg]	71.2 ± 15.0	67.3 ± 14.2	82.1 ± 11.8
Height [cm]	169 ± 10.5	164 ± 6.7	182 ± 8.3
BMI [kg·m-2]	24.8 ± 3.7	24.7 ± 4.0	24.8 ± 3.0
predicted RMR [kcal·min-1]	0.87 ± 0.15	0.80 ± 0.09	1.06 ± 0.06
Resting HR [bpm]	74.2 ± 18.9	75.6 ± 18.8	70.3 ± 19.7
Peak HR [bpm]	141 ± 19.9	142 ± 21.6	139 ± 15.2
Highest VO2 observed during protocol [ml·kg-1·min-1]	23.5 ± 7.5	22.1 ± 6.3	27.4 ± 9.3
Risk of cardiovascular death in following 10 years [% SCORE]	2.6 ± 1.1 (n = 27)	2.4 ± 1.1 (n = 20)	3.3 ± 0.8 (n = 5)
Highest RPE attained during protocol [BORG]	15.9 ± 1.8	15.7 ± 1.6	16.3 ± 2.3
Self-reported time spend in MVPA per week [min/week] (GPAQ)	4866 ± 4666	5184 ± 4916	4230 ± 3936
Physical-activity related health competence [BGK sum score]	39.5 ± 6.9	39.4 ± 6.5	39.7 ± 8.2

BMI: Body Mass Index; VO2 observed during protocol; resting HR: lowest mean heart rate observed over 15 seconds during sitting; RMR: resting metabolic rate predicted with Harrington equation[18]; SCORE: Systematic Coronary Risk Evaluation[16]; RPE: Borg Rating of Perceived Exertion Scale; GPAQ: Global Physical Activity Questionnaire [23]; BGK: Physical Activity-Related Health Competence Questionnaire [24]

^a^Data presented as mean ± standard deviation.

^b^ Demographics of all participants we included in the analysis. Please note that not all participants provide date for four activity trackers.

The ActiGraph data for n = 2 participants and Actiheart data for n = 1 participant were missing due to human error during the initialization process. In addition, the Actiheart data could not be analysed due to signal loss for n = 1 for the complete protocol and for the ergometer test for n = 1. For n = 6, data loss occurred for more than one minute. This period was set as invalid, as described above, and mean was calculated from the remaining data. Out of n = 34, the data of n = 21 was valid for the full protocol, without any lost data. Heart rate data were lost during walking activities (n = 4), household activities (n = 2), or during the ergometer test (n = 1).

One participant did not complete all the tasks of the activity protocol (ergometer data are missing) but fulfilled all other activities, so his/her data are included (see S1 Fig in S1 File for Participant flow chart).

For the total protocol, both the ActiGraph (-64% ± 25%) and the Actiheart (-43% ± 26%) underestimated PAEE in the full sample (n = 32). In a subsample of only women (n = 17), the Fitbit Charge 3 (+78% ± 78%) overestimated and the OMRON Pedometer (-55% ± 47%) underestimated PAEE.

The ActiGraph showed the best estimates for walking activities at different speeds and the biggest difference to IC measured PAEE for household activities and the ergometer protocol. The Actiheart PAEE estimates were closest to IC PAEE for resting activities (-25% ± 14% difference) and walking stairs (-25% ± 37% difference) (see Table 2).

**Table 2:** Physical activity-induced energy expenditure (PAEE) as predicted by branched equation models [11] (group calibration, Actiheart), Freedson algorithms [12] (ActiGraph) compared to PAEE measured by indirect calorimetry during an activity protocol (resting, walking, household activities and cycle ergometer protocol) in older adults with increased cardiac risk (n = 34; n = 25 women). For resting activities, total energy expenditure (TEE) was reported instead of PAEE.

	Measured EE (indirect calorimetry)		HR (Polar belt)	Acceleration (ActiGraph)	HR and acceleration (Actiheart)		Estimated EE		Difference *estimated* minus measured EE	
	PAEE [Kcal/min] or TEE [Kcal/min] for the resting conditions (n = 34)	MET (n = 34)	HR [min^-1^] (n = 34)	ACC [counts· min^-1^] (Vertical axis) (n = 32)	HR [min^-1^] (n = 32)	ACC [counts· min^-1^] (n = 32)	PAEE [Kcal min^-1^] (TEE [Kcal min^-1^] for the resting conditions)		Difference in PAEE [%] (TEE for the resting conditions)	
							ActiGraph (n = 32)	Actiheart (n = 33)	ActiGraph (n = 32)	Actiheart (n = 32)
**Total activity protocol** n = 34	2.24 ± 0.70	2.52 ± 0.44	91.4 ± 16.1 (n = 34)	736 ± 222 (n = 32)	91.9 ± 18.0 (n = 32)	138 ± 42 (n = 32)	0.83 ± 0.63 (n = 32)	1.26 ± 0.59 (n = 32)	- 64.1 ± 24.7 (n = 32)	-42.9 ± 25.6 (n = 32)
**Resting**^**a**^ n = 34	1.36 ± 0.28	1.13 ± 0.19	70.8 ± 17.6 (n = 34)	95 ± 63 (n = 32)	73.6 ± 18.3 (n = 32)	11 ± 7 (n = 32)	0.90 ± 0.19 (n = 32)	1.01 ± 0.24 (n = 32)	- 33.1 ± 11.6 (n = 32)	- 25.0 ± 13.9 (n = 32)
**Walking** n = 34	2.53 ± 0.68	2.83 ± 0.44	91.4 ± 17.2 (n = 34)	2016 ± 718 (n = 32)	90.1 ± 23.3 (n = 30)	388 ± 140 (n = 32)	2.92 ± 1.84 (n = 32)	1.92 ± 1.01 (n = 32)	+ 8.9 ± 55.4 (n = 32)	- 24.6 ± 34.4 (n = 32)
**Stairs** n = 34	3.01 ± 1.14	3.22 ± 0.86	98.5 ± 19.5 (n = 33)	1503 ± 354 (n = 32)	103.0 ± 21.1 (n = 32)	316 ± 94 (n = 32)	1.92 ± 1.23 (n = 32)	2.13 ± 1.18 (n = 32)	- 37.0 ± 41.6 (n = 32)	- 25.2 ± 37.3 (n = 32)
**household** n = 34	2.17 ± 0.53	2.50 ± 0.42	94.3 ± 18.8 (n = 34)	298 ± 158 (n = 32)	94.1 ± 19.7 (n = 32)	89 ± 72 (n = 32)	0.06 ± 0.13 (n = 32)	1.09 ± 0.59 (n = 32)	- 97.8 ± 4.5 (n = 32)	- 49.0 ± 24.0 (n = 32)
**Ergometer (total)** ^**b**^ n = 33	4.32 ± 1.42 (n = 33)	4.20 ± 1.03 (n = 33)	110.8 ± 12.9 (n = 33)	734 ± 1005 (n = 31)	112.3 ± 25.3 (n = 30)	106 ± 83 (n = 30)	1.32 ± 3.54 (n = 31)	2.40 ± 1.53 (n = 30)	-74.1 ± 64.1 (n = 31)	-42.9 ± 37.8 (n = 30)

PAEE: Physical activity induced energy expenditure calculated as daily energy expenditure minus resting metabolic rate as predicted with Harrington Equation[18]; TEE: total energy expenditure calculated as PAEE plus resting metabolic rate predicted with Harrington equation[18]; HR: heart rate; ACC: accelerometer; MET: metabolic equivalent of task;

^a^Changes in position were included in analysis; ^b^Note that 4 out of 33 participants were asked to stop the protocol at BORG 15 instead of total exhaustion because of their cardiac risk.

For total protocol the PAEE estimates with Freedson vector magnitude equation were: 1.31 ± 0.64 kcal/min (- 41.9% ± 22.7% difference to IC) (values not shown in the table)

A one-way ANOVA was performed on n = 15 (participants who provided data for all four devices) and showed significant differences between the PAEE mean values (F (4, 60) = 44.7, p < .05). In addition, the estimated PAEE of all four activity trackers was significantly different from the criterion measure (p < .05). The PAEE estimates of all devices were significantly different from each other (p < .05), with the exception of the PAEE estimated by the Fitbit compared to the PAEE estimated by the ActiGraph (p = .79) and the Actiheart (p = .12).

**Fig 1:**
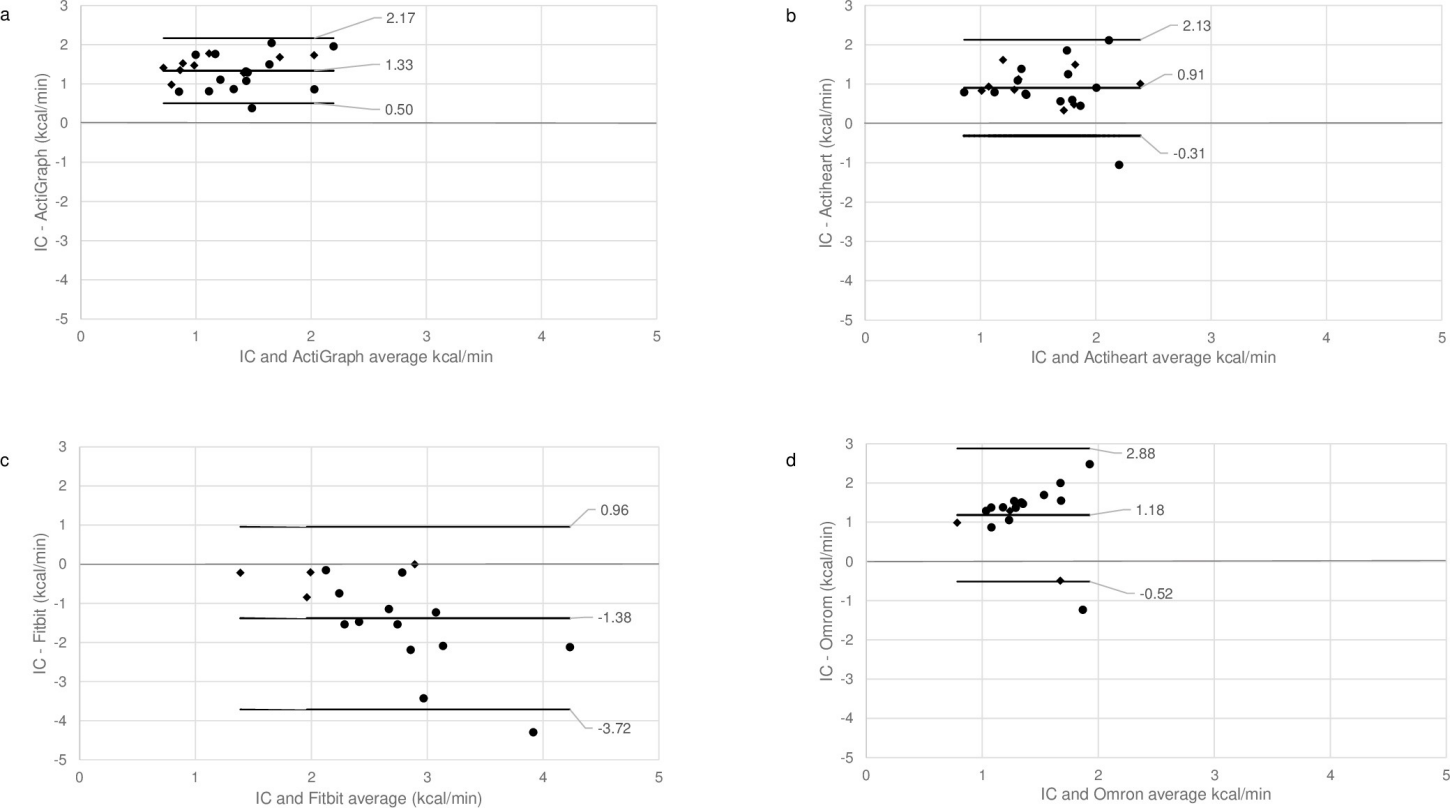
Bland-Altman plots of PAEE estimated by the four activity trackers and by indirect calorimetry. Bland–Altman plots assessing agreement between estimated physical activity induced energy expenditure (PAEE) measured with research and commercially available accelerometers and measured by indirect calorimetry (IC) during an activity protocol (resting, walking, household activities and ergometer protocol) of n = 23 older women with increased cardiac risk: (a) ActiGraph and IC PAEE (kcal/min), b) Actiheart and IC PAEE (kcal/min) and n = 17 older women with increased cardiac risk: c) Fitbit and IC PAEE (kcal/min), and d) OMRON and IC PAEE (kcal/min). Dots represent data from participants where PAEE was measured with all four activity trackers (n = 15), rhombus represent data from participants where PAEE was estimated with two or three activity trackers. Numbers in the Bland–Altman plots are mean difference and upper and lower limits of agreement (95% CI).

For the total study population, Bland–Altman plots showed limits of agreement spanning from +0.05 to +2.82 kcal/min for PAEE estimates from the ActiGraph and -0.31 to +2.32 kcal/min for the Actiheart (see S3 File). In a subsample of only women, Bland–Altman plots showed limits of agreement for PAEE estimates of –3.72 to +0.96 kcal/min and -0.52 to +2.88 kcal/min for the commercially available activity trackers Fitbit Charge 3 and OMRON, respectively (see Fig 1).

## Discussion

Precisely measuring PAEE or EE is essential to assess PA behaviour and to understand the dose–response relationship between PA and CVD. The current study is the first to validate different activity trackers (trackers used in research or commercially available activity trackers) against indirect calorimetry in adults over 60 years, with a special focus on older women with cardiovascular risk performing various activities.

The comparison of EE estimates in the current study demonstrated that the research-grade activity trackers appear to have advantages over the consumer-level activity trackers, even though the estimation of PAEE was not consistently good across the devices, as various activities were measured with varying validity.

Both the research-based activity trackers underestimated PAEE for the total activity protocol ranging from sedentary to moderately vigorous activities, in line with previous research [26]. Our Actiheart estimates of PAEE for the total study population indicate that the simultaneous use of HR and accelerometry using a previously established branched equation model (group-calibrated model) results in a smaller mean prediction error when compared to accelerometer only. However, in the female part of the population, the random error is larger than in the ActiGraph estimates. Thus, the Actiheart does not necessarily provide a more accurate estimate of EE than accelerometry only. Previous studies have reported that the ActiGraph and the Actiheart generally allow a valid estimate of PAEE in several populations [9, 26, 27], while they both tend to underestimate the PAEE induced by most activities.

Considering the activities individually, PAEE of walking activities was generally estimated most accurately, with an especially small mean prediction error by the ActiGraph in women. For PAEE of climbing stairs, there was no difference between ActiGraph and Actiheart estimation accuracy. The ActiGraph seriously underestimated PAEE for household activities and cycling on a stationary bike; the Actiheart performed better for these activities.

Our results indicate that when compared to low-intensity PA, vigorous PA can be captured more accurately by the combined analysis of HR and acceleration data (Actiheart), while the ActiGraph is less accurate at high intensity. This has frequently been reported previously (e.g. Crouter et al. [28]). Previous results from Aguilar-Farias et al. [10] indicate that when estimated by the ActiGraph using the Freedson equation, PAEE tended to be underestimated by about 1.5 MET during higher-intensity activities in older adults. However, generally, most of the activities which older adults undertake are of low intensity.

According to the Bland–Altman plots, too, the scattering of the disagreement between the ActiGraph or the Actiheart and IC seems to be related to PA intensity. For the estimation of PAEE during household activities, heterogeneous results are reported in the literature. While Aguilar-Farias et al. [10] stated that the ActiGraph tended to overestimate EE for lower intensity activities by about 1 MET in older adults, Imboden et al. [14] observed a large underestimation of PAEE during household activities (-26% bias to IC). In line with our results, it can be assumed that for individuals spending more time in sedentary behaviour, light physical activity or walking activities during the day, the ActiGraph will likely yield better measurement accuracy than for individuals spending more time in activities such as cycling or household activities [14].

Despite the Actiheart device showing a more accurate PAEE estimate than the ActiGraph, the signal loss of the heart rate is a serious problem that severely affects the reliability of the PAEE estimate. Thus, adding heart rate may indeed reduce the potential error associated with using a single equation for the acceleration vs. EE relationship, which is derived from just one activity. However, this advantage comes with the caveat that data do not seem to be collected in a reliable fashion, as observed in previous studies [29]. These results may inform researchers who have to select a suitable activity tracker for a specific research question and study design. Researches might prefer to capture a wide variety of activities relatively precisely but at a high risk of signal loss, or alternatively accept a higher underestimation of PAEE while ensuring that the signal is reliably captured.

The accuracy of PAEE estimates by the ActiGraph accelerometer differs depending on the actual activity observed. Physical activity energy expenditure during walking was captured relatively well, in line with previous studies (r = .82) [27]. By contrast, PAEE during the cycle ergometer test and household activities was largely underestimated. In the target population of older adults, walking is a preferred and highly prevalent type of physical activity [30]. Still, older adults, and women more than men, also devote a great deal of time to household activities [31]. The underestimation of PAEE in these activities can be explained for the ActiGraph and the OMRON by the waist placement of the devices. The waist receives little or no vertical acceleration during upper-body movements and physical activities, which do not involve a transfer of the centre of mass.

The extreme interindividual variation of differences between IC- and ActiGraph-PAEE during the cycle ergometer test indicates that in some persons activity is captured relatively well, while in others no acceleration at all is captured. It is well known that accelerometer-based PAEE estimation with standard equations do not produce useful results for cycling activities in older adults (e.g. Kossi et al. [32]: measurement bias 0.81 ≤ ES ≤ 1.90). In fact, the vertical axis counts in our study were surprisingly high, but the additional vector magnitude analysis showed slightly better results.

Overall, our results of the ActiGraph underestimating PAEE can also be found for free-living situations. In adults between 43 and 83 years, PAEE estimated by accelerometer was 50–73% lower than PAEE determined by the DLW method [9].

Of the commercially available activity trackers, the Fitbit Charge 3 overestimated PAEE and show very wide limits of agreement. The Bland-Altman plot may indicate that overestimation of PAEE increases with increasing PA intensity. These findings indicate that, without the GPS, the Fitbit Charge 3 does not produce acceptable estimates, especially at higher self-selected intensities. However, the findings contradict previous findings that the Fitbit Charge devices underestimate PAEE in different age groups [33]. The OMRON underestimated PAEE and showed less wide limits of agreement than the Fitbit, albeit still larger than the research-grade devices. Similarly, it has been reported that the OMRON pedometer underestimated PAEE with limited accuracy [34]. Unlike in our study, in that research the magnitude of underestimation depended on the walking speed. Generally, our results are in good agreement with the review of Fuller et al. [15] who reported that EE estimates of consumer-level activity trackers varied widely, with less than 10% of estimates falling within acceptable limits in controlled settings.

Our sample is as old as, or slightly younger than the population in published ActiGraph validation studies, which however focus on other aspects, such as cut point or equation validation [10, 35]. Their aerobic fitness seems relatively good compared to norm values for 60–69-year-old adults [36], reflecting the problem, that due to the pandemic situation, we had to skip the second part of our study and did not include participants with cardiac disease.

A potential limitation of our study is that the activities were simulated in a laboratory instead of performed in a real-world setting. Participants were encouraged to perform activities as they usually do, but the setting, duration of the activity, measurements (especially the facemask), and the presence of the investigator might have influenced their performance. It could be criticised, that the fact that different activities are grouped into one activity category in our activity protocol means that activity energy is not consistently recorded. In our study, we aimed to estimate the PAEE during short periods of activity followed by breaks, as usually found in analyses of physical activity in the age group and close to daily life (e.g. walking around the house, walking and waiting at traffic lights) [37, 38].

Another limitation concerns the selection of the formulae and algorithms to calculate PAEE, even though we chose the most current default formula (group calibration) [11], cut-points and algorithm [12] for each device to report PAEE as provided in the literature. There are studies evaluating different cut-off values and algorithms for older populations [10, 35], but within the scope of our project we decided to validate the most common ones only. Further studies will focus on the evaluation of other algorithms and cut off-values as, for example metabolic costs increase with age, but our results at least partly suggest that the problem of inaccurate PAEE estimates may only be solved by more advanced technology and intelligent pattern-detecting algorithms.

A strength of this study is its focus on women with a light to moderate cardiovascular risk, an understudied but important group, and directly comparing various devices for a variety of activities of daily living typical of the age group over 60 years against indirect calorimetry. While it may be sufficient for many clinical studies to analyse the criterion validity of steps or time spent in moderate-to-vigorous PA, this study offers a significant advantage in analysing the criterion validity of PAEE.

## Conclusions

The accurate assessment of PAEE in older adults with increased cardiac risk is important in various research and clinical contexts, and is especially understudied in women. In this study, we have directly compared the validity of four different activity trackers against IC.

Overall, research-grade activity trackers provide better validity than consumer-level activity trackers, but seriously underestimate PAEE during cycling and household activities (ActiGraph), or do not reliably capture all signals (Actiheart). Clinicians and researchers should be aware of the potential limitations of these devices.

### Practical implications

- For measuring PAEE in older adults with cardiovascular risk factors, ActiGraph and Actiheart should be preferred over consumer-level activity trackers. However, the risk of signal loss when using the Actiheart should always be considered.
- Physical activity-induced energy expenditure while walking is captured by ActiGraph and Actiheart, with a smaller mean bias in the ActiGraph.
- Physical activity-induced energy expenditure on a stationary bicycle ergometer and during household activities are extremely difficult to assess. If the signal is properly captured, Actiheart PAEE estimates are closer to the criterion measure, whereas the ActiGraph seriously underestimates true PAEE.

## Supporting information

S1 File Supplementary tables and flow of participants. (PDF)

S2 File Supplementary data: PAEE comparison of all activity trackers and indirect calorimetry for subsample (men or women). (PDF)

S3 File Supplementary data: Bland-Altman plots: Total study population. (PDF)

S4 File Raw data set. (XLSX)

## References

[1] Arvidsson D, Fridolfsson J, Börjesson M. Measurement of physical activity in clinical practice using accelerometers. Intern Med J. 2019;286(2):137–53. doi: 10.1111/joim.12908 30993807

[2] Pelliccia A, Sharma S, Gati S, Bäck M, Börjesson M, Caselli S, et al. 2020 ESC Guidelines on sports cardiology and exercise in patients with cardiovascular disease: The Task Force on sports cardiology and exercise in patients with cardiovascular disease of the European Society of Cardcccciology (ESC). Eur Heart J. 2020;42(1):17–96.

[3] Evenson KR, Buchner DM, Morland KB. Objective measurement of physical activity and sedentary behavior among US adults aged 60 years or older. Prev Chronic Dis. 2012;9:E26. 22172193 PMC3277387

[4] Sylvia LG, Bernstein EE, Hubbard JL, Keating L, Anderson EJ. Practical guide to measuring physical activity. J Acad Nutr Diet. 2014;114(2):199–208. doi: 10.1016/j.jand.2013.09.018 24290836 PMC3915355

[5] Wijndaele K, Westgate K, Stephens SK, Blair SN, Bull FC, Chastin SF, et al. Utilization and Harmonization of Adult Accelerometry Data: Review and Expert Consensus. Med Sci Sports Exerc. 2015;47(10):2129–39. doi: 10.1249/MSS.0000000000000661 25785929 PMC4731236

[6] Schrack JA, Cooper R, Koster A, Shiroma EJ, Murabito JM, Rejeski WJ, et al. Assessing Daily Physical Activity in Older Adults: Unraveling the Complexity of Monitors, Measures, and Methods. J Gerontol A Biol Sci Med Sci. 2016;71(8):1039–48. doi: 10.1093/gerona/glw026 26957472 PMC4945889

[7] Montoye AHK, Moore RW, Bowles HR, Korycinski R, Pfeiffer KA. Reporting accelerometer methods in physical activity intervention studies: a systematic review and recommendations for authors. Br J Sports Med. 2018;52(23):1507–16. doi: 10.1136/bjsports-2015-095947 27539504

[8] Hannan AL, Harders MP, Hing W, Climstein M, Coombes JS, Furness J. Impact of wearable physical activity monitoring devices with exercise prescription or advice in the maintenance phase of cardiac rehabilitation: systematic review and meta-analysis. BMC Sports Sci Med Rehabil. 2019;11:14. doi: 10.1186/s13102-019-0126-8 31384474 PMC6668165

[9] Chomistek AK, Yuan C, Matthews CE, Troiano RP, Bowles HR, Rood J, et al. Physical Activity Assessment with the ActiGraph GT3X and Doubly Labeled Water. Med Sci Sports Exerc. 2017;49(9):1935–44. doi: 10.1249/MSS.0000000000001299 28419028 PMC5561512

[10] Aguilar-Farias N, Peeters G, Brychta RJ, Chen KY, Brown WJ. Comparing ActiGraph equations for estimating energy expenditure in older adults. J Sports Sci. 2019;37(2):188–95. doi: 10.1080/02640414.2018.1488437 29912666 PMC6298850

[11] Brage S, Brage N, Franks PW, Ekelund U, Wong MY, Andersen LB, et al. Branched equation modeling of simultaneous accelerometry and heart rate monitoring improves estimate of directly measured physical activity energy expenditure. J Appl Physiol (1985). 2004;96(1):343–51. doi: 10.1152/japplphysiol.00703.2003 12972441

[12] Freedson PS, Melanson E, Sirard J. Calibration of the Computer Science and Applications, Inc. accelerometer. Med Sci Sports Exerc. 1998;30(5):777–81. doi: 10.1097/00005768-199805000-00021 9588623

[13] Nelson MB, Kaminsky LA, Dickin DC, Montoye AH. Validity of Consumer-Based Physical Activity Monitors for Specific Activity Types. Med Sci Sports Exerc. 2016;48(8):1619–28. doi: 10.1249/MSS.0000000000000933 27015387

[14] Imboden MT, Nelson MB, Kaminsky LA, Montoye AH. Comparison of four Fitbit and Jawbone activity monitors with a research-grade ActiGraph accelerometer for estimating physical activity and energy expenditure. Br J Sports Med. 2018;52(13):844–50. doi: 10.1136/bjsports-2016-096990 28483930

[15] Fuller D, Colwell E, Low J, Orychock K, Tobin MA, Simango B, et al. Reliability and Validity of Commercially Available Wearable Devices for Measuring Steps, Energy Expenditure, and Heart Rate: Systematic Review. JMU. 2020;8(9):e18694. 32897239 10.2196/18694 PMC7509623

[16] Piepoli MF, Hoes AW, Agewall S, Albus C, Brotons C, Catapano AL, et al. 2016 European Guidelines on cardiovascular disease prevention in clinical practice: The Sixth Joint Task Force of the European Society of Cardiology and Other Societies on Cardiovascular Disease Prevention in Clinical Practice (constituted by representatives of 10 societies and by invited experts)Developed with the special contribution of the European Association for Cardiovascular Prevention & Rehabilitation (EACPR). Eur Heart J. 2016;37(29):2315–81. 27222591 10.1093/eurheartj/ehw106 PMC4986030

[17] Macfarlane DJ, Wong P. Validity, reliability and stability of the portable Cortex Metamax 3B gas analysis system. Eur J Appl Physiol. 2012;112(7):2539–47. doi: 10.1007/s00421-011-2230-7 22075643 PMC3371330

[18] Harrington M, St. Jeor S, Silverstein L. Predicting resting energy expenditure from body mass index: practical applications and limitations:annual conference proceeding:North American Association for the Study of Obesity. Obesity Research 1997;5:17S.

[19] Pavlidou E, Petridis D, Tolia M, Tsoukalas N, Poultsidi A, Fasoulas A, et al. Estimating the agreement between the metabolic rate calculated from prediction equations and from a portable indirect calorimetry device: an effort to develop a new equation for predicting resting metabolic rate. Nutr Metab (Lond). 2018;15:41. doi: 10.1186/s12986-018-0278-7 29983723 PMC6003108

[20] Weir JB. New methods for calculating metabolic rate with special reference to protein metabolism. J Physiol. 1949;109(1–2):1–9. doi: 10.1113/jphysiol.1949.sp004363 15394301 PMC1392602

[21] Brage S, Brage N, Franks PW, Ekelund U, Wareham NJ. Reliability and validity of the combined heart rate and movement sensor Actiheart. Eur J Clin Nutr. 2005;59(4):561–70. doi: 10.1038/sj.ejcn.1602118 15714212

[22] llgen H, Hansel J. Vorsorgeuntersuchung bei Sporttreibenden: S1-Leitlinie. German Sports Physicians Association (DGSP). 2007.

[23] Armstrong T, Bull F. Development of the World Health Organization Global Physical Activity Questionnaire (GPAQ). J Public Health. 2006;14(2):66–70.

[24] Sudeck G, Pfeifer K. Physical activity-related health competence as an integrative objective in exercise therapy and health sports –conception and validation of a short questionnaire. Ger J Exerc Sport Res. 2016;46(2):74–87.

[25] Altman DG, Bland JM. Measurement in Medicine: The Analysis of Method Comparison Studies. J R Stat Soc Ser D Stat Soc. 1983;32(3):307–17.

[26] Dannecker KL, Sazonova NA, Melanson EL, Sazonov ES, Browning RC. A comparison of energy expenditure estimation of several physical activity monitors. Med Sci Sports Exerc. 2013;45(11):2105–12. doi: 10.1249/MSS.0b013e318299d2eb 23669877 PMC3800491

[27] McMinn D, Acharya R, Rowe D, Gray S, Allan J. Measuring Activity Energy Expenditure: Accuracy of the GT3X+ and Actiheart Monitors. J Exerc Sci. 2013;6(3):217–29.

[28] Crouter SE, Churilla JR, Bassett DR, Jr. Accuracy of the Actiheart for the assessment of energy expenditure in adults. Eur J Clin Nutr. 2008;62(6):704–11. 17440515 10.1038/sj.ejcn.1602766

[29] Júdice PB, Santos DA, Hamilton MT, Sardinha LB, Silva AM. Validity of GT3X and Actiheart to estimate sedentary time and breaks using ActivPAL as the reference in free-living conditions. Gait Posture. 2015;41(4):917–22. doi: 10.1016/j.gaitpost.2015.03.326 25852024

[30] Amireault S, Baier JM, Spencer JR. Physical Activity Preferences Among Older Adults: A Systematic Review. J Aging Phys Act. 2017;27(1):128–39. 10.1123/japa.2017-0234 29283793

[31] Stalling I, Albrecht BM, Doerwald F, Bammann K. Time allocation to active domains, physical activity, and health indicators in older adults: cross-sectional results from the OUTDOOR ACTIVE study. BMC public health. 2020;20(1):1580. doi: 10.1186/s12889-020-09708-z 33081732 PMC7576691

[32] Kossi O, Lacroix J, Ferry B, Batcho CS, Julien-Vergonjanne A, Mandigout S. Reliability of ActiGraph GT3X+ placement location in the estimation of energy expenditure during moderate and high-intensity physical activities in young and older adults. J Sports Sci. 2021:1–8. doi: 10.1080/02640414.2021.1880689 33514289

[33] Chevance G, Golaszewski NM, Tipton E, Hekler EB, Buman M, Welk GJ, et al. Accuracy and Precision of Energy Expenditure, Heart Rate, and Steps Measured by Combined-Sensing Fitbits Against Reference Measures: Systematic Review and Meta-analysis. JMU. 2022;10(4):e35626–e. doi: 10.2196/35626 35416777 PMC9047731

[34] Giannakidou DM, Kambas A, Ageloussis N, Fatouros I, Christoforidis C, Venetsanou F, et al. The validity of two Omron pedometers during treadmill walking is speed dependent. Eur J Appl Physiol. 2012;112(1):49–57. doi: 10.1007/s00421-011-1951-y 21479653

[35] Santos-Lozano A, Santin-Medeiros F, Cardon G, Torres-Luque G, Bailon R, Bergmeir C, et al. Actigraph GT3X: validation and determination of physical activity intensity cut points. Int J Sports Med. 2013;34(11):975–82. doi: 10.1055/s-0033-1337945 23700330

[36] American College of Sports M, Liguori G, Feito Y, Fountaine C, Roy BA. ACSM’s guidelines for exercise testing and prescription. Eleventh edition ed. Philadelphia: Wolters Kluwer Philadelphia; 2022.

[37] Orendurff MS, Schoen JA, Bernatz GC, Segal AD, Klute GK. How humans walk: bout duration, steps per bout, and rest duration. J Rehabil Res Dev. 2008;45(7):1077–89. doi: 10.1682/jrrd.2007.11.0197 19165696

[38] Adams M, Carrascosa L, Jansen CP, Ritter Y, Schwenk M. "Can Do" vs. "Do Do" in Older Adults: A Cross-Sectional Analysis of Sensor-Derived Physical Activity Patterns. Sensors (Basel, Switzerland). 2023;23(4). 10.3390/s23041879 PMC9959454 36850476

