# Supplementary material for: Validation of activity trackers to estimate energy expenditure in older adults with cardiovascular risk factors
Source: PLoS One. 2024 Aug 27;19(8):e0309481. doi: 10.1371/journal.pone.0309481 (PMC11349177; doi:10.1371/journal.pone.0309481)

S1: Supplementary tables and flow of participants

S1 Table 1: Inclusion and Exclusion criteria

**Inclusion**	**Exclusion**
sufficient German language skills (written and spoken)	instable cardiovascular disease, without medical permission for physical activity or medical release for an ergometer test[a].
live independently at home or were currently undergoing inpatient care or inpatient or outpatient rehabilitation	Missing medical preliminary investigation in the event of unclear health status or existing indications for relative contraindications.
	Serious illness (e.g. tumors, fractures, acute infections, central neurological disorders, cognitive impairments) where in this specific situation physical activity is contraindicated or participation is unsafe
	severe physical immobility and limitation of mobility where support for climbing stairs, as well as for standing up or sitting down (stair, stationary bicycle ergometer) was needed
	severe neurodermatitis or other severe skin diseases
	serious acute infections
	impairment of wound healing or severe sensibility disturbances of the legs (diabetic foot) caused by diabetes mellitus type II
	No medical permission for physical activity or load test
	Contraindications for physical activity according to recommendations from professional societies as ACC/AHA guidelines (Arnett et al., 2019).

[a]Participants with an increased cardiac risk needed a Physician's permission in order to perform ergometer test on the bicycle

S1 Table 2: Full activity protocol consisting of simulated activities of daily living and a cycle ergometer test.

Activity	Description of activity	Compendium METs	Compendium Code (2011)	time (in min)
	Sedentary (5 tasks)			
Sitting	Seated, quietly, avoid bodily movement	1.3	07021	3
Sitting and talking	Seated, free talking	1,5	09055	1
Lying down	Lying in supine position, avoid bodily movement	1.3	07011	3
Standing	Standing on the floor, avoid bodily movement	1.3	07040	3
Standing and talking	Standing, free talking	1,8	09050	1
	Indoor Walking (resting between changes of walking speed for 30seconds) (4 tasks)			
Slow walking (self-paced)	Strolling	2.0	17151	1,5
Walking with habitual walking speed (self-paced)	Normal walking	3.5	17160	1,5
Walking with habitual walking speed with bags (self-paced)	Normal walking with two 2kg bags	4,8	21065	1,5
Brisk walking (self-paced)	Walking as fast as possible without running	5.0	17220	1,5
Rest	Sitting quietly	1.3	07021	2
	Stairs climbing (2 tasks)			
Climbing up the stairs	Walking up the stairs at self-selected pace	4.0	17133	1
Rest	Standing quietly	1.3	07040	1
Climbing down the stairs	Walking down the stairs at self-selected pace	3.5	17070	1
Rest	Sitting quietly	1.3	07021	2
	Activities of daily living (5 tasks)			
Cleaning a table	Standing, cleaning a table	2.3	05011	1,5
Sweeping the floor	Standing, sweeping the floor with broom and hand brush	3.3	05010	1,5
Laundry	Carrying towels from laundry rack, standing and folding towels	2.3	05095	1,5
Desk work	Sitting, sorting folders	1.5	11580	1,5
Window cleaning	Standing, window cleaning	3.2	05022	1,5
Rest	Sitting quietly	1.3	07021	2-5
	Cycle ergometer protocol	25 W / 2 minutes		

S1 Figure 1: Flow of participants

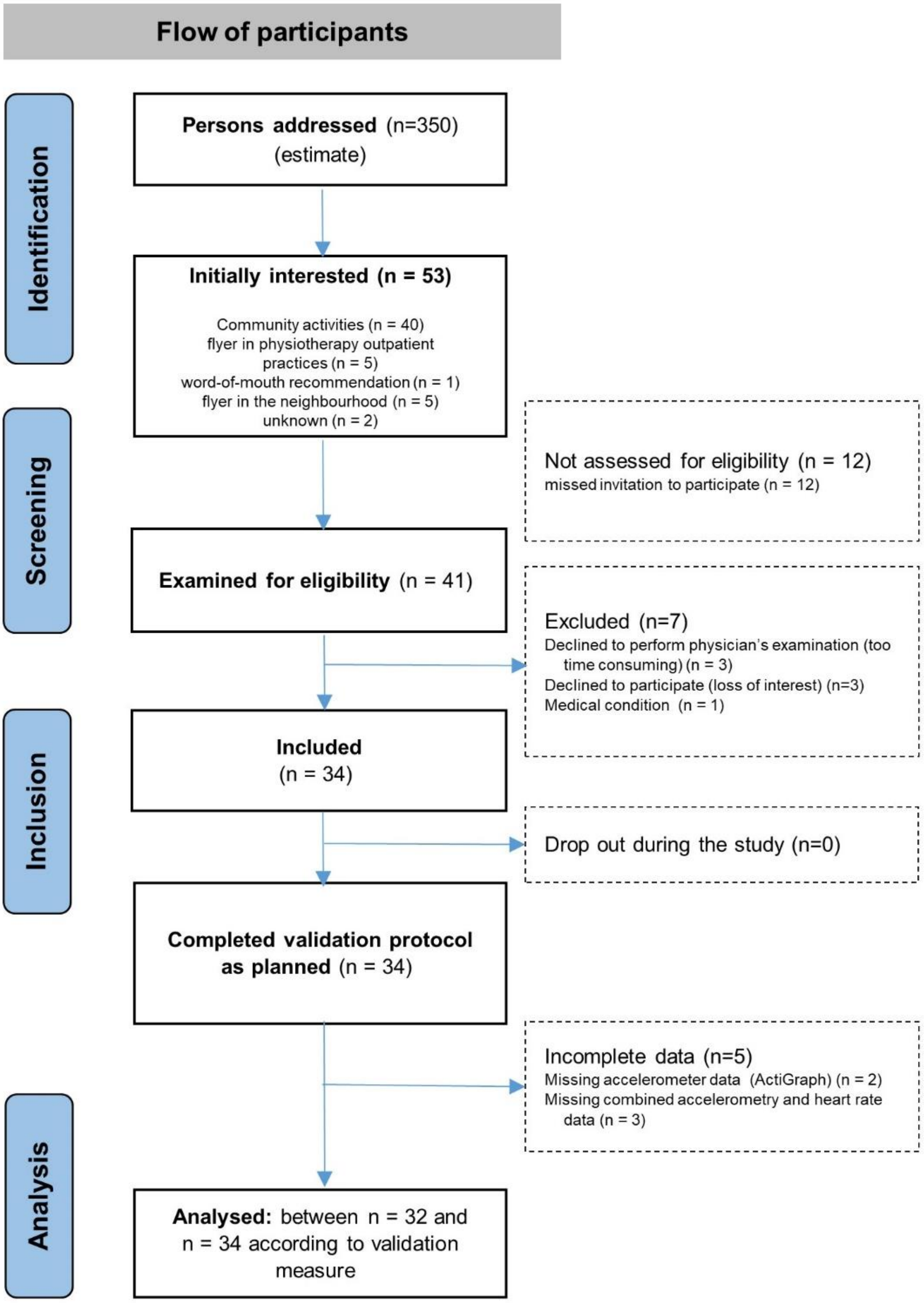

Supplement: S1 File — (PDF) [file pone.0309481.s001.pdf]
