# Supplementary material for: Validation of activity trackers to estimate energy expenditure in older adults with cardiovascular risk factors
Source: PLoS One. 2024 Aug 27;19(8):e0309481. doi: 10.1371/journal.pone.0309481 (PMC11349177; doi:10.1371/journal.pone.0309481)
Supplement: S2 File — (PDF) [file pone.0309481.s002.pdf]

S2 Supplementary data: PAEE comparison of all activity trackers and indirect calorimetry for subsample (men or women)

S2 Table 1: Physical activity-induced energy expenditure (PAEE) as predicted by branched equation models (group calibration, Actiheart) (Brage et al., 2004), algorithms (ActiGraph) (Freedson et al., 1998) or calculated with the specific software provided by the manufacturer (OMRON, Fitbit) compared to PAEE measured by indirect calorimetry during an activity protocol (resting, walking, household activities, and cycle ergometer protocol) in older women with increased cardiac risk (n = 25). For resting activities, total energy expenditure (TEE) was reported instead of PAEE. Note that the number of participants equipped with the Fitbit wristwatch and the OMRON pedometer is smaller than the number of participants observed with the ActiGraph accelerometer and the Actiheart ECG and activity recorder.

	**Measured EE (indirect calorimetry)**		**HR (Polar belt)**	**Acceleration (ActiGraph)**	**HR and acceleration (Actiheart)**		**Fitbit**	**OMRON**	**Estimated EE**				**Difference** ***estimated*** **minus measured EE**			
									PAEE [Kcal min^{-1}] (TEE for the resting conditions)				Difference in PAEE [%] (TEE for the resting conditions)			
	PAEE [Kcal/min] TEE [Kcal/min] for the resting conditions (n=25)	MET (n=25)	HR [min^{-1}] n=25	ACC [counts·min-1] (Vertical axis) (n=23)	HR [min^{-1}] (n=23)	ACC [counts·min^{-1}] (n=23)	Steps (n=17)	Steps (n=17)	ActiGraph n=23	Actiheart n=23	Fitbit n=17	OMRON n=17	ActiGraph n=23	Actiheart n=23	Fitbit n=17	OMRON n=17
**Total activity protocol**	2.0 ± 0.5 (n=25)	2.4 ± 0.4 (n=25)	94.1 ± 17.5 (n=25)	679 ± 228 (n=23)	95.3 ± 18.9 (n=23)	140 ± 48 (n=23)	1822 ± 256 (n=17)	1555 ± 290 (n=17)	0.66 ± 0.43 (n=23)	1.1 ± 0.6 (n=23)	3.4 ± 1.2 (n=17)	0.77 ± 0.56 (n=17)	- 68.0 ± 19.0 (n=23)	- 43.2 ± 28.9 (n=23)	+ 77.6 ± 77.7 (n=17)	- 55.1 ± 47.4 (n=17)

**Resting**[a]	1.3 ± 0.2 (n=25)	1.1 ± 0.2 (n=25)	75.6 ± 18.8 (n=25)	161 ± 111 (n=23)	69 ± 46 (n=23)	11 ± 8 (n=23)	-	-	0.8 ± 0.1 (n=23)	0.9 ± 0.2 (n=23)	-	-	- 34.2 ± 12.7 (n=23)	- 25.6 ± 15.6 (n=23)	-	-
**Walking**	2.4 ± 0.6 (n=25)	2.8 ± 0.5 (n=25)	94.7 ± 17.3 (n=25)	1942 ± 706 (n=23)	96.4 ± 19.3 (n=23)	363 ± 146 (n=23)	-	-	2.45 ± 1.39 (n=23)	1.7 ± 1.0 (N=23)	-	-	- 0.5 ± 45.6 (n=23)	- 28.9 ± 39.1 (n=23)	-	-
**Stairs**	3.2 ± 1.3 (n=25)	3.5 ± 0.9 (n=25)	103.1 ± 19.2 (n=25)	1459 ± 469 (n=23)	108.6 ± 21.6 (n=23)	323 ± 107 (n=23)	-	-	1.6 ± 1.2 (n=23)	2.2 ± 1.4 (n=23)	-	-	- 56.5 ± 19.7 (n=23)	-31.6 ± 36.9 (n=23)	-	-
**household**	2.1 ± 0.5 (n=25)	2.5 ± 0.5 (n=25)	98.7 ± 18.5 (n=25)	282 ± 142 (n=23)	99.8 ± 20.3 (n=23)	106 ± 78 (n=23)	-	-	0.1 ± 0.1 (n=23)	1.1 ± 0.6 (n=23)	-	-	- 98.2 ± 3.6 (n=23)	- 47.0 ± 25.1 (n=23)	-	-
**Ergometer (total)** [b]	3.9 ± 1.0 *(n=24)*	4.1 ± 0.8 (n=24)	113.2 ± 12.8 (n=24)	652 ± 1037 (n=22)	119.1 ± 14.4 (n=21)	122 ± 84 (n=21)	-	-	0.6 ± 1.8 (n=22)	2.4 ± 1.6 (n=21)	-	-	- 83.2 ± 52.2 (n=22)	- 40.9 ± 43.7 (n=21)	-	-

PAEE: Physical activity-induced energy expenditure calculated as daily energy expenditure minus resting metabolic rate as predicted with Harrington equation; TEE: total energy expenditure calculated as PAEE plus resting metabolic rate predicted with Harrington equation; HR: heart rate; ACC: accelerometer; MET: metabolic equivalent of task

[a]Changes in position were included in analysis; [b]Note that 4 out of 24 participants were asked to stop the protocol at BORG 15 instead of total exhaustion because of their cardiac risk.

S2 Table 2: Physical Activity induced energy expenditure (PAEE) as predicted by branched equation models (group calibration, Actiheart) (Brage et al., 2004), Freedson (1998) algorithms (ActiGraph) compared to PAEE measured by indirect calorimetry during the cycle ergometer protocol (25 W / 2 min) in older women with cardiac risk (n = 24 conducted the protocol).

	**Measured EE (indirect calorimetry)**		**HR (Polar belt)**	**Acceleration (ActiGraph)**	**HR and acceleration (Actiheart)**		**Estimated EE**		**Difference *estimated* minus measured EE**	
	PAEE [Kcal/min] (n=25)	MET (N=25)	HR [min^{-1}] N=25	ACC [$counts \cdot min^{-1}$] (Vertical axis) (n=23)	HR [min^{-1}] (n=23)	ACC [$counts \cdot min^{-1}$] (n=23)	PAEE [Kcal min^{-1}]		Difference in PAEE [%]	
							ActiGraph	Actiheart	ActiGraph	Actiheart
**25W**	2.3 ± 0.5 (n=24)	2.8 ± 0.6 (n=25)	96.5 ± 10.8 (n=24)	568 ± 1018 (n=22)	100.5 ± 12.1 (n=21)	85 ± 76 (n=21)	0.6 ± 1.8 (n=22)	1.1 ± 0.5 (n=21)	- 74.4 ± 76.1 (n=22)	- 51.5 ± 21.2 (n=21)
**50W**	3.5 ± 0.5 (n=24)	3.8 ± 0.7 (n=25)	109.6 ± 11.4 (n=24)	593 ± 1049 (n=22)	116.6 ± 14.3 (n=21)	81 ± 52 (n=21)	0.6 ± 1.8 (n=22)	1.6 ± 0.8 (n=21)	- 83.8 ± 49.8 (n=22)	- 52.7 ± 21.3 (n=21)

**75W**	4.7 ± 0.6 (n=20)	4.7 ± 0.9 (n=20)	123.2 ± 14.2 (n=20)	588 ± 793 (n=18)	129.7 ± 16.2 (n=17)	121 ± 101 (N=17)	0.4 ± 1.2 (n=18)	2.8 ± 1.4 (n=17)	- 92.0 ± 25.1 (n=18)	- 41.0 ± 28.7 (n=17)
**100W**	6.2 ± 0.8 (n=11)	5.9 ± 1.0 (n=11)	136.5 ± 11.0 (n=11)	960 ± 974 (n=9)	143.0 ± 10.8 (n=11)	181 ± 164 (n=11)	0.8 ± 1.7 (n=9)	4.0 ± 2.1 (n=11)	-87.4 ± 25.0 (n=9)	-34.4 ± 34.9 (n=11)
**125W**	7.1 ± 0.8 (n=7)	6.5 ± 1.4 (n=7)	144.6 ± 9.8 (n=7)	975 ± 934 (n=5)	146.9 ± 12.4 (n=7)	176 ± 212 (n=7)	0.8 ± 1.8 (n=5)	4.8 ± 2.8 (n=7)	- 90.3 ± 21.6 (n=5)	-32.1 ± 39.9 (n=7)
**150 W[a]**	9.5 ± 0.0 (n=1)	5.4 ± 0.0 (n=1)	143.4 (n=1)	391 (n=1)	148.6 ± 0.0 (n=1)	86 (n=1)	0.0 (n=1)	4.1 (n=1)	- 100 (n=1)	-57.3 (n=1)

PAEE: Physical activity-induced energy expenditure calculated as daily energy expenditure minus resting metabolic rate as predicted with Harrington equation; TEE: total energy expenditure calculated as PAEE plus resting metabolic rate predicted with Harrington equation; HR: heart rate; ACC: accelerometer; MET: metabolic equivalent of task

[a]Note that 4 out of 24 participants were asked to stop the protocol at BORG 15 instead of total exhaustion because of their cardiac risk.

S2 Table 3: Physical Activity induced energy expenditure (PAEE) as predicted by branched equation models (group calibration, Actiheart) (Brage et al., 2004), Freedson (1998) algorithms (ActiGraph) compared to PAEE measured by indirect calorimetry during an activity protocol (resting, walking, household activities and cycle ergometer protocol) in older men (n = 9). For resting activities, total energy expenditure (TEE) was reported instead of PAEE.

	**Measured EE (indirect calorimetry)**		**HR (Polar belt)**	**Acceleration (ActiGraph)**	**HR and acceleration (Actiheart)**		**Estimated EE**		**Difference *estimated* minus measured EE**	
	PAEE [Kcal/min] TEE [Kcal/min] for Resting (n=9)	MET (n=9)	HR [min^{-1}] (n=9)	ACC [counts·min^{-1}] (Vertical axis) (n=9)	HR [min^{-1}] (n=9)	ACC [counts·min^{-1}] (n=9)	PAEE [Kcal min^{-1}] (TEE for the resting conditions)		Difference in PAEE [%] (TEE for the resting conditions)	
							ActiGraph (n=9)	Actiheart (n=9)	ActiGraph (n=9)	Actiheart (n=9)
**Total activity protocol**	3.0 ± 0.7	2.8 ± 0.5	85.9 ± 11.0	881 ± 253	86.9 ± 11.2	133 ± 44	1.3 ± 0.9	1.7 ± 0.5	- 54.1 ± 34.9	-40.7 ± 15.5
**Resting [a]**	1.7 ± 0.2	1.2 ± 0.1	70.3 ± 19.7	159 ± 55	66.1 ± 9.3	12 ± 5	1.2 ± 0.2	1.3 ± 0.1	- 30.3 ± 7.9	- 22.6 ± 8.6
**Walking**	3.0 ± 0.6	2.9 ± 0.3	82.2 ± 13.8	2203 ± 981	86.3 ± 14.1	452 ± 175	4.1 ± 2.4	2.7 ± 0.6	+33.0 ± 72.7	-11.3 ± 10.7

**Stairs**	2.5 ± 0.3	2.5 ± 0.3	84.1 ± 12.1 (n=8)	1617 ± 298	88.9 ± 11.8	296 ± 103	2.8 ± 1.0	2.3 ± 0.8	+12.6 ± 42.2	- 6.3 ± 33.2
**household**	2.4 ± 0.6	2.5 ± 0.3	82.3 ± 14.4	339 ± 212	83.7 ± 14.9	45 ± 19	0.1 ± 0.2	1.2 ± 0.7	- 96.9 ± 6.4	-52.8 ± 21.8
**Ergometer (total)**	5.4 ± 1.9	4.6 ± 1.5	104.5 ± 11.5	934 ± 906	108.6 ± 12.7	68 ± 68	3.0 ± 5.8	2.9 ± 1.3	- 52.0 ± 86.4	-45.1 ± 20.1

PAEE: Physical activity-induced energy expenditure calculated as daily energy expenditure minus resting metabolic rate as predicted with Harrington equation; TEE: total energy expenditure calculated as PAEE plus resting metabolic rate predicted with Harrington equation; HR: heart rate; ACC: accelerometer; MET: metabolic equivalent of task

[a]Changes in position were included in analysis;

S2 Table 4: Physical Activity induced energy expenditure (PAEE) as predicted by branched equation models (group calibration, Actiheart) (Brage et al., 2004), Freedson (1998) algorithms (ActiGraph) compared to PAEE measured by indirect calorimetry during the cycle ergometer protocol (25 W / 2 min) in older men (n = 9).

	**Measured EE (indirect calorimetry)**		**HR (Polar belt)**	**Acceleration (ActiGraph)**	**HR and acceleration (Actiheart)**		**Estimated EE**		**Difference *estimated* minus measured EE**	
	PAEE [Kcal/min]	MET	HR [min^{-1}]	ACC [counts·min^{-1}] (Vertical axis)	HR [min^{-1}]	ACC [counts·min^{-1}]	PAEE [Kcal min^{-1}]		Difference in PAEE [%]	
							ActiGraph	Actiheart	ActiGraph	Actiheart
**25W**	2.2 ± 0.6 (n=9)	2.4 ± 0.4 (n=9)	82.5 ± 13.5 (n=9)	610 ± 794 (n=9)	84.4 ± 13.4 (n=9)	28 ± 27 (n=9)	0.6 ± 1.9 (n=9)	0.9 ± 0.6 (n=9)	- 74.3 ± 77.1 (n=9)	- 57.5 ± 21.2 (n=9)
**50W**	3.1 ± 0.7 (n=9)	3.0 ± 0.6 (n=9)	89.3 ± 15.7 (n=9)	708 ± 849 (n=9)	91.9 ± 15.0 (n=9)	32 ± 35 (n=9)	0.8 ± 2.4 (n=9)	1.3 ± 0.8 (n=9)	- 75.9 ± 72.4 (n=9)	- 60.9 ± 24.1 (n=9)
**75W**	4.4 ± 0.8 (n=9)	3.9 ± 0.8 (n=9)	98.1 ± 17.9 (n=9)	847 ± 934 (n=9)	101.2 ± 16.8 (n=9)	41 ± 49 (n=9)	0.9 ± 2.8 (n=9)	1.9 ± 0.8 (n=9)	- 79.3 ± 62.1 (n=9)	- 57.0 ± 15.9 (n=9)
**100W**	5.6 ± 0.8 (n=9)	4.7 ± 1.0 (n=9)	107.5 ± 17.7 (n=9)	926 ± 1080 (n=9)	111.1 ± 17.1 (n=9)	53 ± 54 (n=9)	1.00 ± 2.9 (n=9)	2.7 ± 1.0 (n=9)	- 83.2 ± 50.4 (n=9)	- 51.3 ± 17.4 (n=9)

**125W**	6.8 ± 1.0 (n=8)	5.6 ± 1.3 (n=8)	112.7 ± 12.7 (n=8)	1155 ± 1071 (n=8)	117.4 ± 13.6 (n=8)	72 ± 71 (n=8)	1.4 ± 3.0 (n=8)	3.6 ± 1.3 (n=8)	- 79.3 ± 44.6 (n=8)	- 46.9 ± 19.6 (n=8)
**150 W**	9.0 ± 0.3 (n=5)	7.3 ± 1.0 (n=5)	123.0 ± 17.0 (n=5)	1141 ± 602 (n=5)	127.4 ± 17.8 (n=5)	144 ± 132 (n=5)	0.7 ± 1.5 (n=5)	5.4 ± 2.7 (n=5)	- 92.9 ± 15.9 (n=5)	- 38.8 ± 30.9 (n=5)
**175 W**	10.4 ± 0.7 (n=5)	8.3 ± 1.2 (n=5)	133.8 ± 18.1 (n=5)	1347 ± 693 (n=5)	138.7 ± 17.0 (n=5)	202 ± 160 (n=5)	1.4 ± 2.9 (n=5)	6.5 ± 2.8 (n=5)	- 87.0± 27.1 (n=5)	- 37.3 ± 25.8 (n=5)
**200 W**	12.1 ± 0.5 (n=3)	8.7 ± 1.1 (n=3)	133.9 ± 15.8 (n=3)	1388 ± 990 (n=3)	138.9 ± 13.2 (n=3)	207 ± 120 (n=3)	2.3 ± 4.0 (n=3)	6.4 ± 2.1 (n=3)	- 81.6 ± 32.0 (n=3)	- 47.6 ± 17.3 (n=3)
**225 W**	13.8 ± 1.0 (n=2)	10.0 ± 0.4 (n=2)	135.4 ± 12.1 (n=2)	1325 ± 103 (n=2)	133.8 ± 18.7 (n=2)	199 ± 209 (n=2)	1.2 ± 1.7 (n=2)	6.68 ± 4.1 (n=2)	- 91.9 ± 11.5 (n=2)	- 50.4 ± 32.9 (n=2)

PAEE: Physical activity-induced energy expenditure calculated as daily energy expenditure minus resting metabolic rate as predicted with Harrington equation; TEE: total energy expenditure calculated as PAEE plus resting metabolic rate predicted with Harrington equation; HR: heart rate; ACC: accelerometer; MET: metabolic equivalent of task

S2 Table 5: Physical activity-induced energy expenditure (PAEE) as predicted by branched equation models (group calibration, Actiheart) (Brage et al., 2004), Freedson (1998) algorithm (ActiGraph) compared to PAEE measured by indirect calorimetry during the cycle ergometer protocol (25 W / 2min) in older adults (n = 33; n = 24 women).

	**Measured EE (indirect calorimetry)**		**HR (Polar belt)**	**Acceleration (ActiGraph)**	**HR and acceleration (Actiheart)**		**Estimated EE**		**Difference *estimated* minus measured EE** [Kcal min^{-1}]	
	PAEE [Kcal/min]	MET	HR [min^{-1}]	ACC (counts·min^{-1}) (Vertical axis))	HR [min^{-1}]	ACC [counts· min^{-1}]	PAEE [Kcal min^{-1}]		Difference in PAEE [%]	
							ActiGraph	Actiheart	ActiGraph	Actiheart
**25W**	2.2 ± 0.5 (n=33)	2.7 ± 0.6 (n=33)	92.7 ± 13.0 (n=33)	580 ± 958 (n=31)	95.7 ± 14.4 (n=30)	68 ± 70 (n=30)	0.6 ± 1.8 (n=31)	1.1 ± 0.5 (n=30)	- 74.4 ± 75.1 (n=31)	- 53.6 ± 23.8 (n=31)
**50W**	3.4 ± 0.6 (n=33)	3.6 ± 0.8 (n=33)	104.0 ± 15.5 (n=33)	626 ± 995 (n=31)	105.7 ± 16.9 (n=30)	66 ± 52 (n=30)	0.7 ± 2.0 (n=31)	1.5 ± 0.8 (n=30)	- 81.5 ± 56.1 (n=31)	- 55.7 ± 22.0 (n=30)
**75W**	4.6 ± 0.7 (n=29)	4.5 ± 0.9 (n=29)	115.4 ± 19.1 (n=29)	675 ± 841 (n=27)	119.9 ± 21.2 (n=26)	93 ± 94 (n=26)	0.6 ± 1.8 (n=27)	2.5 ± 1.3 (n=26)	- 87.8 ± 40.5 (n=27)	- 46.5 ± 25.8 (n=26)

**100W**	5.9 ± 0.9 (n=20)	5.3 ± 1.1 (n=20)	123.4 ± 20.3 (n=20)	943 ± 1001 (n=18)	128.6 ± 21.4 (n=20)	123 ± 140 (n=20)	0.9 ± 2.3 (n=18)	3.4 ± 1.8 (n=20)	- 85.3 ± 38.6 (n=18)	- 42.0 ± 29.0 (n=20)
**125W**	7.0 ± 0.9 (n=15)	6.0 ± 1.4 (n=15)	127.6 ± 19.8 (n=15)	1085 ± 987 (n=13)	131.2 ± 19.8 (n=15)	120 ± 157 (n=15)	1.2 ± 2.5 (n=13)	4.1 ± 2.1 (n=15)	- 83.5 ± 36.7 (n=13)	- 40.0 ± 30.6 (n=15)
**150 W**	9.1 ± 0.3 (n=6)	7.0 ± 1.2 (n=6)	126.4 ± 17.3 (n=6)	1016 ± 620 (n=6)	130.9 ± 18.1 (n=6)	134 ± 121 (n=6)	0.6 ± 1.4 (n=6)	5.2 ± 2.4 (n=6)	- 94.1 ± 14.5 (n=6)	- 41.9 ± 28.7 (n=6)
**175 W**	10.4 ± 0.7 (n=5)	8.3 ± 1.2 (n=5)	133.8 ± 18.1 (n=5)	1347 ± 693 (n=5)	138.7 ± 17.0 (n=5)	202 ± 153 (n=5)	1.4 ± 2.9 (n=5)	6.5 ± 2.8 (n=5)	- 87.0 ± 27.1 (n=5)	- 37.3 ± 25.8 (n=5)
**200 W**	12.1 ± 0.5 (n=3)	8.7 ± 1.1 (n=3)	133.9 ± 15.8 (n=3)	1388 ± 990 (n=3)	138.9 ± 13.2 (n=3)	207 ± 75 (n=3)	2.3 ± 4.0 (n=3)	6.4 ± 2.1 (n=3)	- 81.6 ± 32.0 (n=3)	- 47.6 ± 17.3 (n=3)
**225 W** [a]	13.8 ± 1.0 (n=2)	10.0 ± 0.4 (n=2)	135.4 ± 12.1 (n=2)	1325 ± 103 (n=2)	133.8 ± 18.7 (n=2)	199 ± 247 (n=2)	1.2 ± 1.7 (n=2)	6.7 ± 4.1 (n=2)	- 91.9 ± 11.5 (n=2)	- 50.4 ± 32.9 (n=2)

PAEE: Physical activity-induced energy expenditure calculated as daily energy expenditure minus resting metabolic rate as predicted with Harrington equation; TEE: total energy expenditure calculated as PAEE plus resting metabolic rate predicted with Harrington equation; HR: heart rate; ACC: accelerometer; MET: metabolic equivalent of task

[a]Note that 4 out of 33 participants were asked to stop the protocol at BORG 15 instead of total exhaustion because of their cardiac risk.
