# Supplementary material for: Validation of activity trackers to estimate energy expenditure in older adults with cardiovascular risk factors
Source: PLoS One. 2024 Aug 27;19(8):e0309481. doi: 10.1371/journal.pone.0309481 (PMC11349177; doi:10.1371/journal.pone.0309481)

S 3: Supplementary data: Bland-Altman plots: total study population

S3 Figure 1: Bland–Altman plots assessing agreement between estimated physical activity-induced energy expenditure (PAEE) measured with research-grade accelerometers (ActiGraph and Actiheart) and measured by indirect calorimetry (IC) during an activity protocol (resting, walking, household activities and ergometer protocol) for the total study population (n = 34, n = 25 women): (a) ActiGraph and IC PAEE (kcal/min), b) Actiheart and IC PAEE (kcal/min). Numbers in the Bland–Altman plots are mean difference and upper and lower limits of agreement (95% CI).

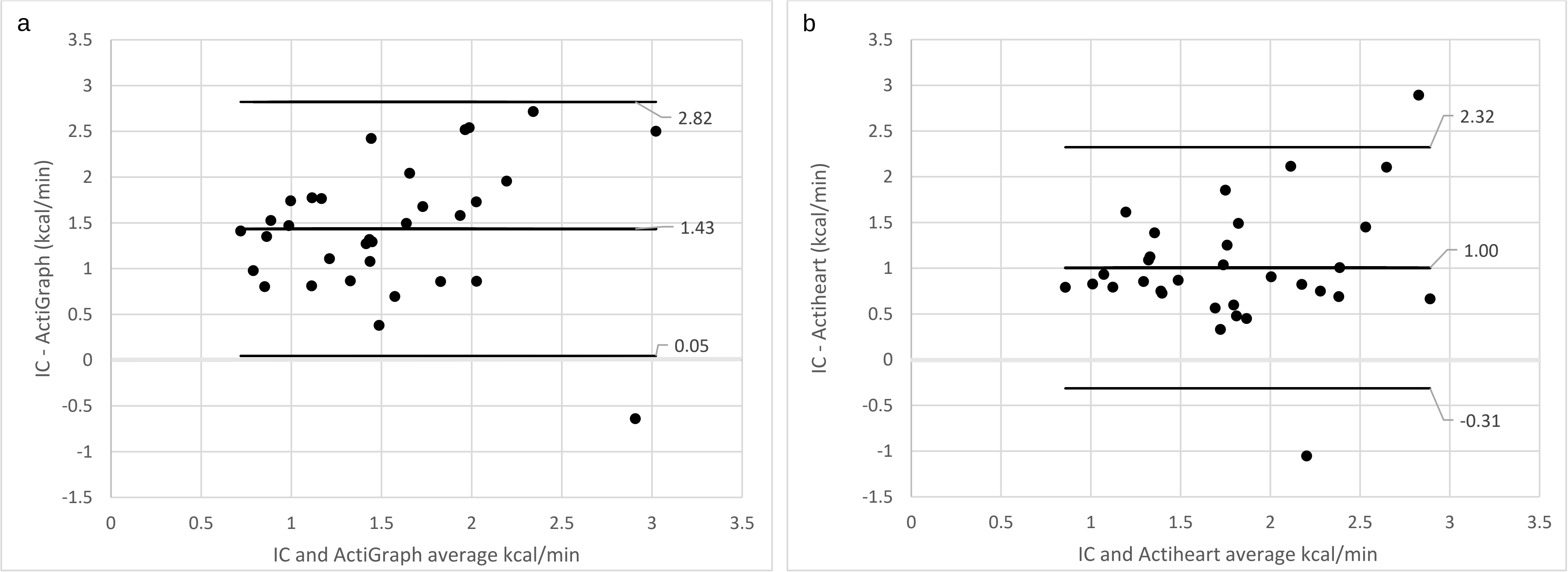

Supplement: S3 File — (PDF) [file pone.0309481.s003.pdf]
